# *Toxoplasma gondii* Toxolysin 4 Contributes to Efficient Parasite Egress from Host Cells

**DOI:** 10.1128/mSphere.00444-21

**Published:** 2021-06-30

**Authors:** My-Hang Huynh, Marijo S. Roiko, Angelica O. Gomes, Ellyn N. Schinke, Aric J. Schultz, Swati Agrawal, Christine A. Oellig, Travis R. Sexton, Jessica M. Beauchamp, Julie Laliberté, Komagal Kannan Sivaraman, Louis B. Hersh, Sheena McGowan, Vern B. Carruthers

**Affiliations:** a Department of Microbiology and Immunology, University of Michigan Medical School, Ann Arbor, Michigan, USA; b Department of Molecular and Cellular Biochemistry and Center for Structural Biology, University of Kentucky, Lexington, Kentucky, USA; c Infection and Immunity Program, Biomedicine Discovery Institute, Department of Microbiology, Monash University, Clayton, Victoria, Australia; d Department of Cardiology, University of Kentucky, Lexington, Kentucky, USA; Indiana University School of Medicine

**Keywords:** *Toxoplasma gondii*, apicomplexan parasites, egress, intracellular parasites, proteases

## Abstract

Egress from host cells is an essential step in the lytic cycle of T. gondii and other apicomplexan parasites; however, only a few parasite secretory proteins are known to affect this process. The putative metalloproteinase toxolysin 4 (TLN4) was previously shown to be an extensively processed microneme protein, but further characterization was impeded by the inability to genetically ablate *TLN4*. Here, we show that TLN4 has the structural properties of an M16 family metalloproteinase, that it possesses proteolytic activity on a model substrate, and that genetic disruption of *TLN4* reduces the efficiency of egress from host cells. Complementation of the knockout strain with the *TLN4* coding sequence significantly restored egress competency, affirming that the phenotype of the Δ*tln4* parasite was due to the absence of TLN4. This work identifies TLN4 as the first metalloproteinase and the second microneme protein to function in T. gondii egress. The study also lays a foundation for future mechanistic studies defining the precise role of TLN4 in parasite exit from host cells.

**IMPORTANCE** After replicating within infected host cells, the single-celled parasite Toxoplasma gondii must rupture out of such cells in a process termed egress. Although it is known that T. gondii egress is an active event that involves disruption of host-derived membranes surrounding the parasite, very few proteins that are released by the parasite are known to facilitate egress. In this study, we identify a parasite secretory protease that is necessary for efficient and timely egress, laying the foundation for understanding precisely how this protease facilitates T. gondii exit from host cells.

## INTRODUCTION

Egress by apicomplexan parasites, including Toxoplasma gondii and malaria parasites (*Plasmodium* spp.), is the critical last step in the lytic cycle. Egress liberates the parasite for infection of new cells and releases host cell cytosolic contents, which can activate an inflammatory immune response (1). Inflammation is a hallmark of toxoplasmosis, with associated ocular, neural, cardiac, or respiratory disease, which is especially severe in immunodeficient or congenitally infected individuals. Severe inflammation during toxoplasmosis is linked to the parasite genotype, with highly virulent strains being associated with worse outcome, especially for ocular and congenital infection (2).

Recent focus on egress is elucidating signaling pathways and associated secondary messengers, providing a broader understanding of the intricate interplay between the signals involved in this complex cascade of events (recently reviewed in reference 3). Such studies have solidified roles for cyclic GMP (cGMP) and calcium to stimulate protein kinase G (PKG) (4, 5) and calcium-dependent protein kinases (CDPKs) (4, 6–8), among other targets. Calcium signaling results in the activation of parasite motility and the discharge of apical secretory granules, termed micronemes (9–12). While the importance of microneme proteins, such as transmembrane adhesins that connect with the actin-myosin motor system to drive gliding motility and active cell invasion, have been established, microneme proteins also contribute to active egress via their role in the formation of pores such as those created by Perforin-like protein 1 (PLP1) (13–15). Gene knockout (KO) studies suggest that PLP1 pore formation disrupts the parasitophorous vacuole membrane (PVM), which encases parasites during intracellular replication. *PLP1*-deficient parasites are delayed or fail in egress and show a marked loss of virulence in infected mice, implying a link between efficient egress and virulence. Although several proteins released from parasite-dense granules (calcium-independent secretory organelles released during parasite replication) have also been implicated in egress (16–20), PLP1 is the only microneme protein known to directly function in egress to date.

Previous work identified a putative metalloprotease, toxolysin 4 (TLN4; TGME49_206510), in a proteomic screen of *Toxoplasma* secretory products released by extracellular tachyzoites (21). A subsequent study showed that TLN4 is a microneme protein that undergoes extensive proteolytic processing and potentially contributes to parasite fitness, based on loss of *TLN4*-deficient parasites from a mixed population of parasites transfected with a knockout plasmid (22). However, a recent genome-wide CRISPR/Cas9 knockout screen indicated that TLN4 does not contribute substantially to parasite fitness based on its phenotype score of 0.51 (on a scale of −7 to 3, with lower values indicating lower fitness) (23). TLN4 is a member of the M16 metalloproteinase subfamily of so-called cryptases, which are represented throughout the tree of life and include four TLN genes in the *Toxoplasma* genome. M16 metalloproteinases are exemplified by the human insulin-degrading enzyme (IDE) and feature a catalytic chamber (or crypt) that accommodates small polypeptides for degradation. Whereas characterization of TLN2 (TGME49_227948) and TLN3 (TGME49_257010) has not been reported, TLN1 (TGME49_269885) resides in the parasite secretory rhoptries, where it plays an unknown role apart from contributing modestly to growth *in vitro* (24).

Here, we show that TLN4 has the structural features of an active metalloproteinase and that it possesses proteolytic activity against a model polypeptide substrate. We also report that *TLN4*-deficient parasites show normal gliding motility, cell invasion, and replication but have a delayed induced-egress phenotype. Our findings suggest that TLN4 contributes to *Toxoplasma* egress, identifying it as only the second microneme protein implicated in this event.

## RESULTS

### TLN4 has the structural features of an active metalloproteinase.

**FIG 1:**
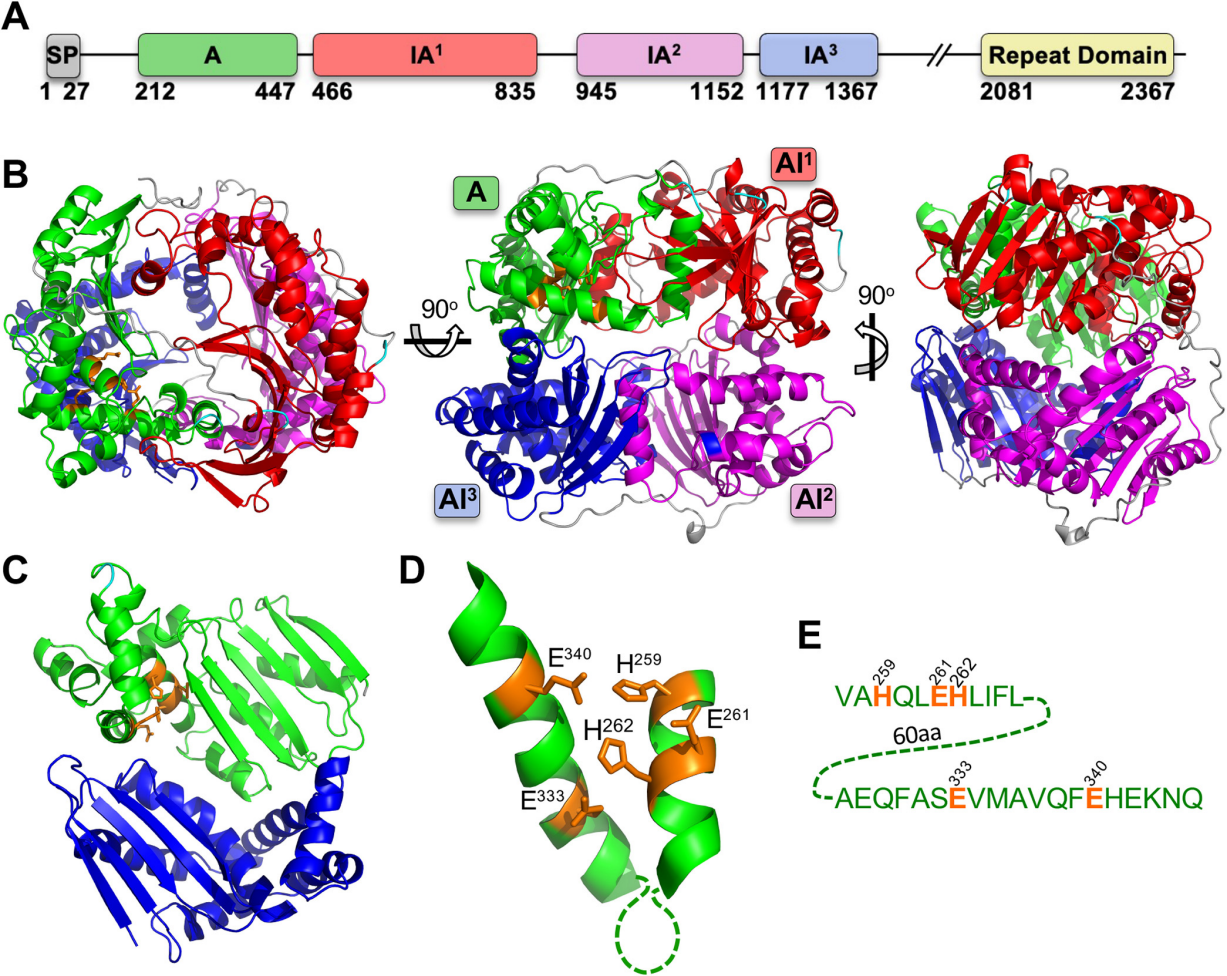
Structural features of TLN4. (A) Domain structure of TLN4 illustrating its possession of a signal peptide (SP) and, based on the IDE structure, a single active domain (domain A), 3 inactive domains (IA^1-3^), and a C-terminal repeat domain. Note that a largely featureless region between IA^3^ and the repeat domain is not shown and is instead denoted by //. Numbering indicates amino acid positions and is based on the sequenced cDNA of TLN4 (22). (B) Structural model of TLN4 showing the arrangement of domains into a shell surrounding a central catalytic chamber. Three different views are provided. (C) The A and AI^3^ domains viewed from the center of the chamber with resides that coordinate zinc binding shown in orange. (D) Zoom-in of the adjacent alpha helices of the A domain showing the residues (orange) that are predicted to coordinate binding to zinc. (E) Sequence of the alpha helices shown in panel D, including residues (orange) that likely mediate binding to zinc as a cofactor for proteolytic activity.

TLN4 is a large (2,435-amino-acid [aa]) protein that contains multiple domains, including one active (A) and three inactive (IA) M16 proteinase domains, along with a long C-terminal extension that includes a repeat domain consisting of eight tandem repeats of a 28-aa sequence (Fig. 1A). Structural modeling of the M16 proteinase domains (residues 202 to 1367) using the human insulin-degrading enzyme as a template suggests that each domain forms a similar αβ roll fold, with the domains arranged around a central chamber (Fig. 1B), as expected for an M16 family metalloproteinase (25). When viewed from a perspective of inside the chamber or crypt, a putative active site is visible (Fig. 1C) with the characteristic HXXEHX_N_EX_6_E binding motif for catalytic Zn^2+^ arranged on two α-helices (Fig. 1D) that are separated by 60 amino acids in TLN4 (Fig. 1E). These features are consistent with TLN4 being an active metalloproteinase of the M16 family.

### Recombinant TLN4 is capable of processing β-insulin.

**FIG 2:**
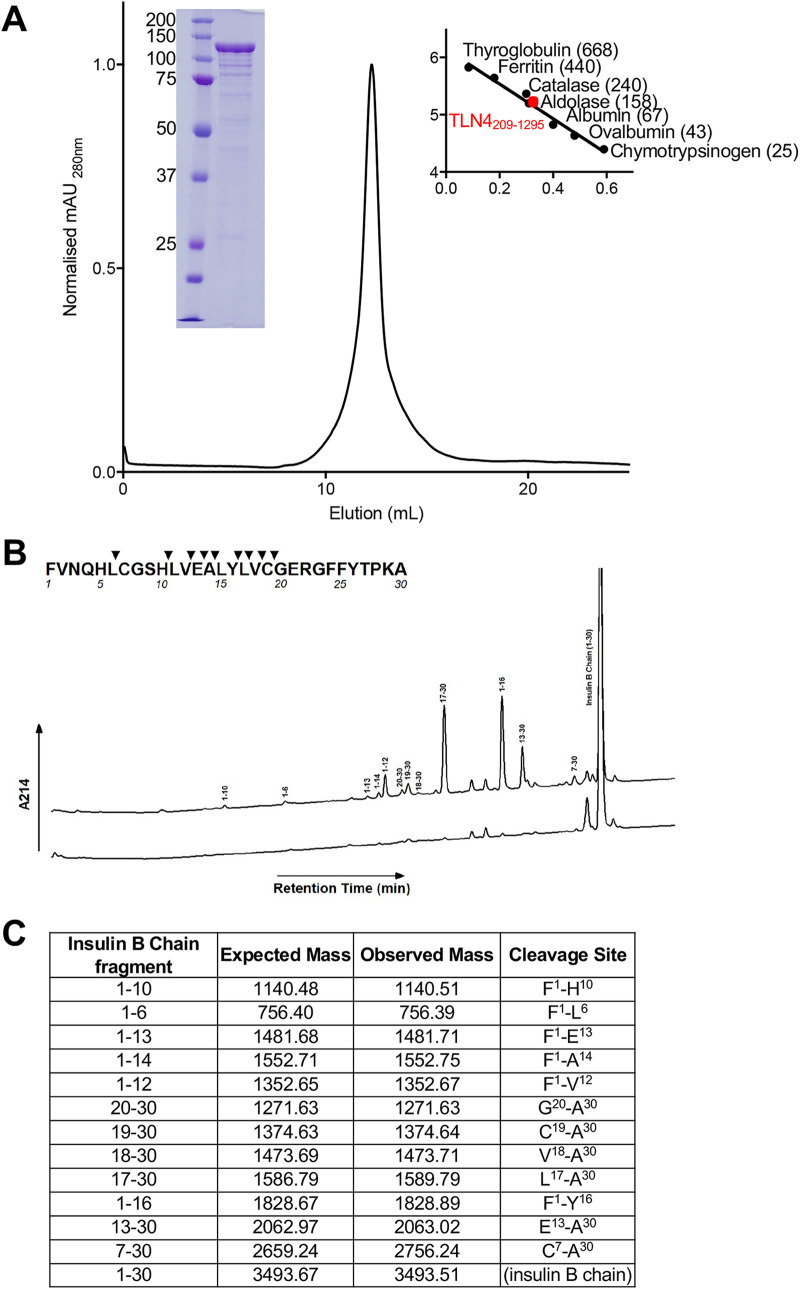
Recombinant TLN4 proteolytic activity using *β-insulin* as a model substrate. (A) Chromatogram showing the elution of recombinant TLN4 as a monodispersed peak from size exclusion chromatography. SDS-PAGE of purified TLN4 is shown inset on the left, with molecular weight markers indicated on the right of the elution peak. Values in parentheses indicate molecular size in kDa. (B) HPLC profile of insulin B chain cleaved by TLN4. Hydrolysis products were determined by comparing a reaction allowed to incubate (top line) versus a reaction that was immediately stopped (bottom line). (Inset) Captured peaks are labeled with their residue number, and a cleavage map was developed. (C) Mass spectrometry analysis of captured hydrolysis products from insulin B chain are shown with their expected mass and observed mass along with the fragment and cleavage sites that each product represents.

M16 family proteinases typically act upon peptides and polypeptides that are sufficiently small to fit in the crypt. To determine if TLN4 possesses proteolytic activity, we expressed and purified a recombinant form of TLN4 lacking the C-terminal extension. TLN4_209–1295_ was expressed using a bacterial expression system and was purified and refolded. The purified protein migrated on SDS-PAGE as a prominent 130-kDa band and behaved as a monodispersed protein when analyzed by size exclusion chromatography (Fig. 2A). Upon incubating recombinant TLN4 with β-insulin as a model substrate, we observed the generation of several cleavage products (Fig. 2B), which were confirmed by mass spectrometry to originate from β-insulin (Fig. 2C). Mapping of the cleavage sites on the sequence of β-insulin revealed a preference for TLN4 cleavage in the central region of the polypeptide but no obvious preference for recognition of specific amino acids. Collectively, our findings suggest that TLN4 is an active protease that is capable of processing a model substrate.

### *TLN4* is amenable to genetic disruption.

**FIG 3:**
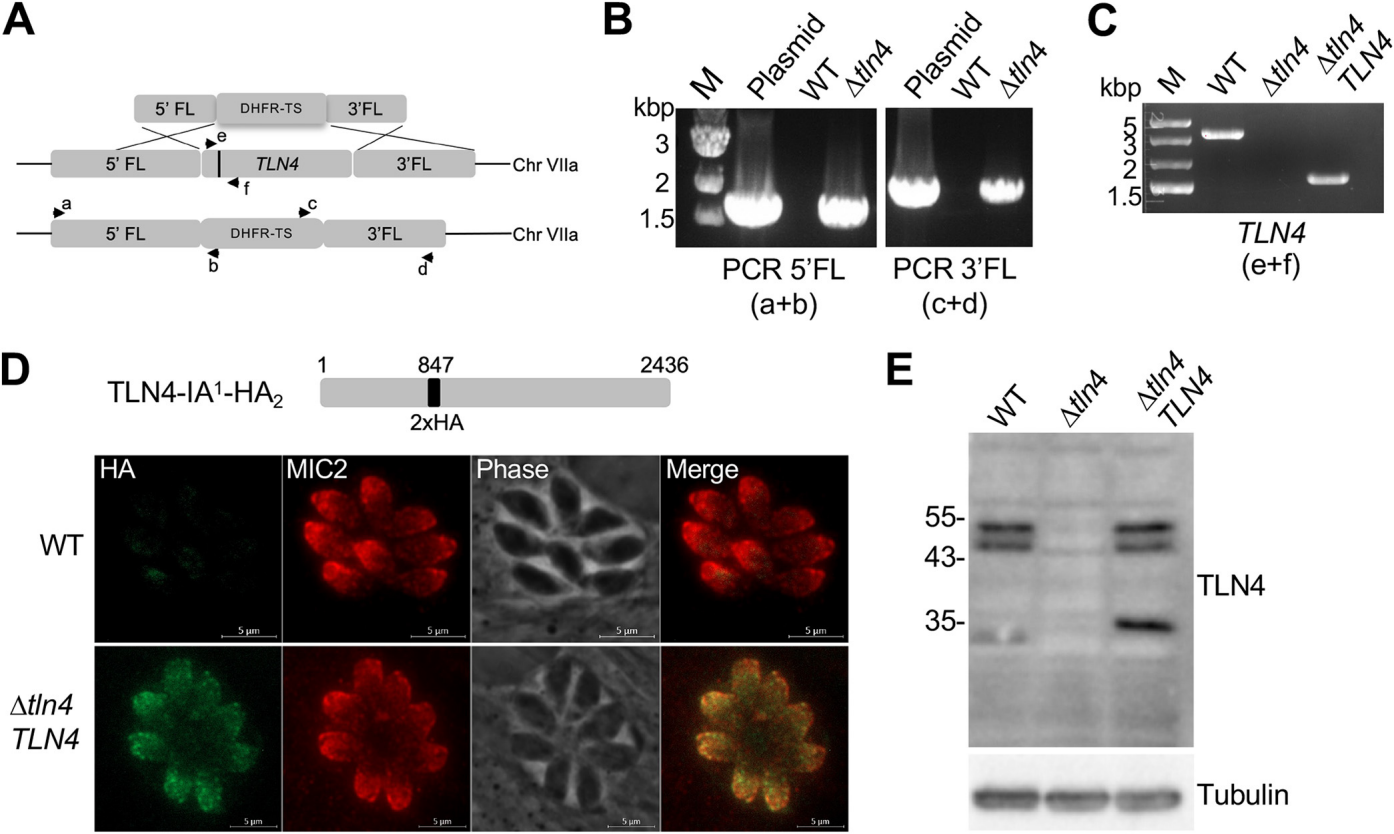
Genetic disruption of *TLN4*. (A) Schematic showing the TLN4 genomic locus and homologous recombination with a cassette containing a *TLN4* 5′ flank, the HXGPRT selectable marker, and a *TLN4* 3′ flank. Arrows and letters indicate the placement of primers used in panel B, and the black bar in TLN4 indicates an intron that permits distinction of the gene from the cDNA in panel C. Other introns of TLN4 are not shown. (B) PCR validation of selectable marker integration at the 5′ (primers a and b) and 3′ (primers c and d) end. M, molecular weight marker. (C) PCR validation of *TLN4* deletion (Δ*tln4*) and complementation (Δ*tln4TLN4*) with the cDNA (primers e and f). The differences in size are due to the presence of an intron in the WT genomic DNA versus the cDNA in the complement strain lacking the intron. (D) Schematic showing the complementation construct with a 2× HA tag in the full-length TLN4 coding sequence. The immunofluorescence panel shows the micronemal localization of the complemented strain, costained with anti-MIC2. (E) Mouse antibodies to TLN4 detect the ∼55-kDa doublet and the ∼32 kDa of TLN4 in the lysates of WT and Δ*tln4TLN4* parasites. Slower mobility of the smallest band in the complement strain is due to the HA tag. Sizes (in kilodaltons) of molecular weight markers are shown to the left. Detection of T. gondii tubulin was included as a loading control.

In a previous study of TLN4, it was proposed that TLN4 contributes to parasite fitness, because *TLN4*-knockout parasites could be detected in a population of transfected RH parasites but were lost through further culturing (22). To enhance the recovery of knockout parasites, we utilized RHΔ*ku80*Δ*hxg* parasites (wild type [WT] here) and transfected them with a hypoxanthine xanthine guanine phosphoribosyl transferase (HXGPRT) selectable cassette flanked by 5′ and 3′ *TLN4* homology regions (Fig. 3A). Individual knockout parasite clones were tested by PCR for integration of the selectable marker at the 5′ and 3′ ends (Fig. 3B) and for the absence of the *TLN4* gene (Δ*tln4*) (Fig. 3C). We then genetically complemented Δ*tln4* parasites by expression of the *TNL4* cDNA containing two copies of a hemagglutinin (HA) epitope tag inserted at aa 847 after the first inactive domain (TLN4-IA^1^-HA_2_) to generate Δ*tln4TLN4* parasites, which were confirmed by PCR (Fig. 3C). The difference in fragment size in the WT and Δ*tln4TLN4* parasites is due to the presence of introns in the WT but not in the cDNA of the complement strain. Expression of TLN4 in Δ*tln4TLN4* parasites was confirmed by fluorescence microscopy (Fig. 3D). We further validated expression by Western blotting, which showed the expected doublet of processed TLN4 at ∼50 kDa and a smaller ∼35-kDa species (Fig. 3E). This smaller species showed the expected increase in size in Δ*tln4TLN4* due to the HA tag. The loss of the TLN4 protein in Δ*tln4* was also confirmed by Western blotting (Fig. 3E).

### TLN4 does not contribute to invasion, replication, gliding motility, or virulence.

**FIG 4:**
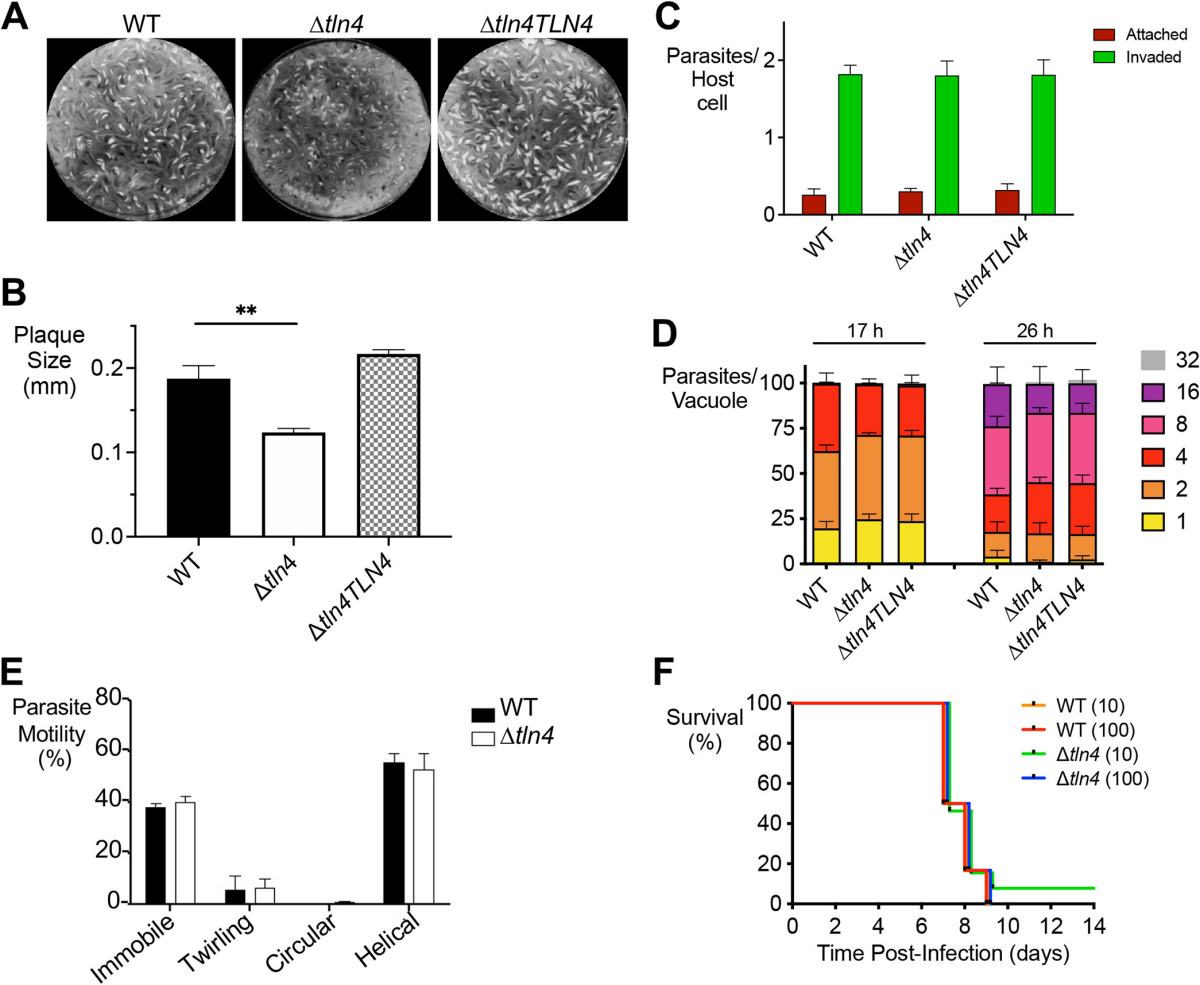
Effect of TLN4-deficient parasites on the lytic cycle. (A) Plaque assays show smaller plaques in Δ*tln4* parasites. One hundred parasites of each strain were inoculated into 6-well plates and allowed to grow for 7 days undisturbed. Wells were stained with crystal violet. (B) Individual plaque sizes were measured using ImageJ. Data represent three independent biological experiments with triplicate samples within each experiment. A minimum of 500 plaques were counted per strain. Error bars represent standard errors of the means (SEM), and statistics were performed using an unpaired, two-tailed, Student's *t* test. (C) Parasites lacking TLN4 show normal invasion. HFF cells in 8-well chamber slides were inoculated with parental, Δ*tln4*, or Δ*tln4TLN4* parasites and allowed to invade for 20 min prior to fixation. Wells were differentially stained with SAG1 antibodies to detect attached or invaded parasites per host cell nucleus. A minimum of 250 host cells were counted per strain. Data in the invasion (C) and replication (D) graphs represent means ± SEM from three independent experiments, each with triplicate samples. At least 8 fields of view were counted per well. (D) Parasites lacking *TLN4* replicate normally. Parasites were inoculated into 8-well chamber slides and allowed to replicate for 17 h or 26 h prior to fixation and enumeration of parasites per vacuole. A minimum of 250 vacuoles were counted per strain per time point. (E) Δ*tln4* parasites show normal modes of gliding motility. Video microscopy to enumerate the types of gliding motility shows the percentages of immobile parasites and parasites performing twirling, circular, and helical gliding in all strains tested. Graphs indicate the means ± SEM from three independent experiments. *, *P ≤ *0.05 by Student's *t* test. (F) TLN4 does not play a role in acute virulence. Swiss-Webster mice were infected intraperitoneally with 10 or 100 tachyzoites of WT or Δ*tln4* parasites, and survival time was enumerated; 12 mice were infected for each strain and inoculum.

To assess whether TLN4 plays a role in the lytic cycle, plaque assays were performed. Compared to parental parasites, Δ*tln4* parasites showed significantly smaller plaques (Fig. 4A and B), suggesting a defect in one or more steps in the parasite lytic cycle. This phenotype was fully restored in the complemented strain. We next tested each step in the lytic cycle to identify the basis of smaller plaques. Parasites deficient in TLN4 showed normal invasion (Fig. 4C) and replication at 17 h and 26 h postinfection (Fig. 4D), and all types of gliding motility were observed (Fig. 4E). Finally, no significant differences were observed in mouse survival of acute infection upon intraperitoneal infection with 10 or 100 tachyzoites (Fig. 4F).

### Δ*tln4* parasites are defective in efficient egress.

**FIG 5:**
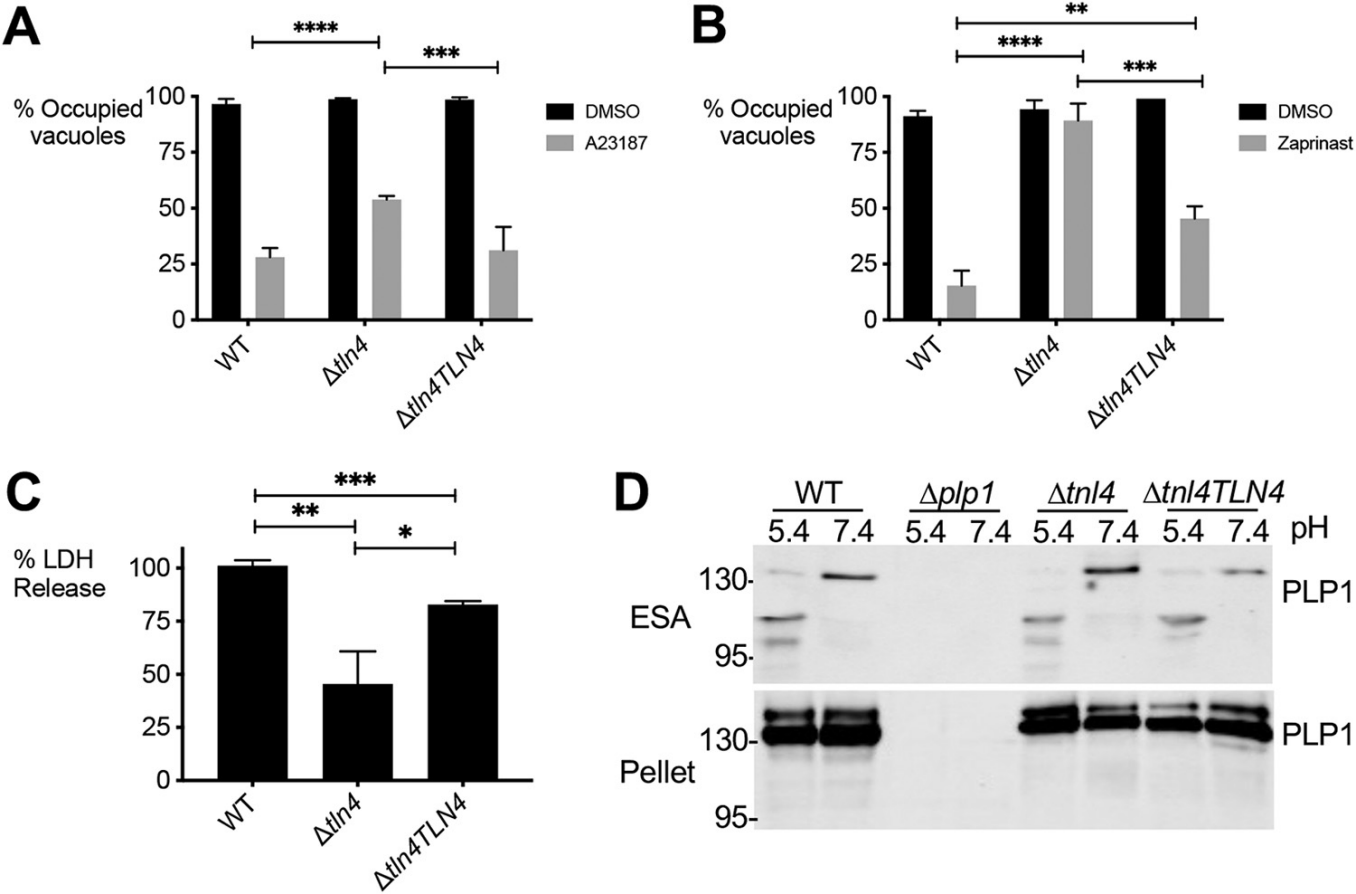
*TLN4*-deficient parasites are defective in egress. (A and B) Parasite egress was quantified by immunofluorescence microscopy. Thirty-hour vacuoles were treated with DMSO or 2 μM A23187 (A) or 200 μM zaprinast (B) for 5 min prior to fixation and enumeration. *, *P ≤ *0.05; **, *P* ≤ 0.01; ***, *P* ≤ 0.001; ****, *P ≤ *0.0001; all by Student's *t* test. A minimum of 500 vacuoles were counted per strain. (C) Lactate dehydrogenase (LDH) release following induction with 100 μM zaprinast was used as a measure of egress. Data are normalized to WT LDH release. All graphs shown are means ± SEM from three biological replicates, each with triplicate samples. (D) Parasites lacking TLN4 have normal PLP1 processing. Pellet and ESA were collected from parasites at pH 5.4 and 7.4, separated on SDS-PAGE, blotted onto membranes, and probed with rabbit anti-PLP1 antibodies. Processing of PLP1 in the ESA of all strains expressing PLP1 observed at pH 5.4. The pellet blot (bottom) acts as a loading control.

The last step of the lytic cycle is egress of the intracellular parasites from host cells, which can be induced by addition of the calcium ionophore A23187. Whereas most wild-type parasites egressed from host cells within 2 min of ionophore addition, at which time the cells were fixed, significantly fewer Δ*tln4* parasites egressed in the allotted time (Fig. 5A). This phenotype is mostly reversed in Δ*tln4TLN4* parasites. The deficiency in egress was more pronounced when zaprinast, a phosphodiesterase inhibitor, was used as an inducer (Fig. 5B), which may be due to its mode of action. Whereas A23187 elevates parasite cytosolic calcium by mobilizing it from parasite and host intracellular stores and the media, zaprinast treatment triggers the release of calcium from intracellular stores via activation of protein kinase G and inositol triphosphate signaling (4, 26). In this regard, zaprinast treatment likely better mimics the physiologic release of Ca^2+^ from intracellular stores during natural egress. An alternative and complementary measure of parasite egress is the release of host lactate dehydrogenase (LDH) into the surrounding medium due to loss of host cell integrity. After normalizing LDH release to WT parasites, Δ*tln4* parasites showed ∼45% release of LDH, while the complement strain nearly restored LDH release to wild-type levels (Fig. 5C). In examining the excreted-secreted antigen fraction of extracellular parasites, conducted in buffers with neutral or acidic pH (to mimic PV acidification [13–15]), there was no change in the processing of PLP1 in the Δ*tln4* parasites compared to WT parasites (Fig. 5D). Together, these findings suggest a role for TLN4 in T. gondii egress from host cells that is independent of PLP1 processing.

## DISCUSSION

TLN4 was previously shown to be an extensively processed microneme protein (22), but its contribution to the lytic cycle was not determined due to the inability to generate a knockout of this gene in the wild-type RH strain background. In this study, we were able to isolate a *TLN4* knockout strain by utilizing the more genetically tractable RHΔ*ku80* strain. Plaque assays showed that Δ*tln4* parasites affected the lytic cycle, based on the smaller plaque sizes observed. Assessing the known steps of the lytic cycle showed that there were no defects in invasion, replication, or types of gliding motility performed by the parasites, and there was no effect on parasite virulence. The only deficiency observed was in induced egress, assessed by a static egress assay, and LDH release. Complementation of Δ*tln4* with a TLN4 construct under the control of the endogenous TLN4 promoter fully restored the plaque defect and largely restored the egress deficiency.

*In vitro* investigation into TLN4 suggests that the protein is an active protease based on cleavage of β-insulin as a model substrate, supporting a potential proteolytic function within the parasite. Homology modeling indicates that TLN4 adopts the typical M16 structure and contains a catalytic chamber or “crypt,” which is one of the defining features of the M16 family of metalloproteinases. Studies with other M16 enzymes indicate that the crypt encapsulates and cleaves polypeptides, with the substrate being determined by the size and charge of the crypt as well as the flexibility of the substrate(s) (27, 28). Although it is possible that TLN4 plays a direct role in egress by, e.g., facilitating the disruption of the PVM, an indirect contribution of TLN4 to egress is also plausible. An indirect role could involve proteolytically activating another parasite protein that contributes to egress or degrading a protein that would otherwise compromise egress. Identifying substrates of TLN4 in future studies might reveal the basis for its contribution to parasite exit from host cells.

PLP1 is the only microneme protein identified to date to have a direct role in egress. Interestingly, mice infected with Δ*tln4* have no virulence defect, whereas Δ*plp1* parasites are markedly virulence attenuated. This notable distinction could be because the egress defect of Δ*tln4* parasites is less pronounced than that for Δ*plp1*, or it could be due to additional roles for PLP1 during infection of mice. Future studies identifying other secretory proteins that contribute to egress to various degrees along with other work defining how PLP1 shapes the outcome of infection should help distinguish between these possibilities.

Among the Apicomplexa, the M16A subfamily is constrained to coccidian parasites, including close (e.g., *Hammondia hammondia* and Neospora caninum) and more distant (*Sarcocystis neurona* and *Eimeria* spp.) relatives of T. gondii (https://orthomcl.org/orthomcl/app). Whereas the genome of T. gondii encodes 11 M16 metalloproteinases, of which 4 belong to the M16A subfamily (29), the genome of the intestinal parasite Cryptosporidium parvum encodes an expanded family of 22 M16 metalloproteinases, 18 of which are M16A members. Among these, INS1 was recently shown to be necessary for formation of macrogamonts, the female sexual stage of C. parvum (30). The results of reciprocal BLAST searches indicate that TLN4 is most closely related to C. parvum INS-15 (cgd3_4260) and INS-16 (cgd3_4270), which are closely related orthologs (83% identical) (30, 31). INS-15 localizes to the mid-apical region of C. parvum sporozoites and possibly merozoites (31). Antibodies to INS-15 impaired sporozoite cell invasion, suggesting a role in parasite entry into host cells. Future studies involving the targeted deletion of M16A family members in C. parvum and other coccidian parasites will be necessary to appreciate further their contributions to parasite infection biology.

## MATERIALS AND METHODS

### Structural modeling of TLN4.

The full amino acid sequence of ME49 strain TLN4 was used to query Phyre2, which identified human insulin-degrading enzyme (PDB entry 2JBU) as the top-scoring template. This analysis resulted in a structural model of TLN4 encompassing amino acids 202 to 1367. The structural model was visualized using PyMOL v2.3.2.

### Expression and purification of recombinant TLN4.

A region encoding domains A to IA-3 of the *TLN4* cDNA from RH strain (amino acids 209 to 1295) was amplified with TLN4.625.NdeI.F (5′-GGAATTCCATATGAGAGACACGAGCGCGTACTCGG-3′) and TLN4.3885.Not.R (5′-AAGGAAAAAAGCGGCCGCGCTAAGCCACCGGAGGCGCTCGAGGAAAGC-3′) primers and cloned into the bacterial expression vector pET21a, introducing an in-frame C-terminal hexahistidine tag to the construct. The Escherichia coli expression construct containing the *TLN4_209–1295_* open reading frame (ORF) was verified by DNA sequencing.

The *TLN4_209–1295_* expression plasmid was transformed into Escherichia coli BL21 Gold cells selected on 2× yeast-tryptone (2YT) broth-agar plates containing 100 μg/ml ampicillin. Twenty milliliters of 2YT supplemented with ampicillin was grown to confluence overnight and then added to 1 liter of 2YT containing ampicillin and incubated with shaking at 37ºC until an optical density at 600 nm (OD_600_) of ∼0.85 was reached. Cultures were then induced with a final concentration of 1 mM isopropyl-β-d-thiogalactopyranoside (IPTG) overnight at 16ºC.

Harvest and cell lysis of the overnight expression culture showed that TLN4_209–1295_ expressed to high yields but was insoluble. Inclusion bodies were prepared by harvest of the insoluble fraction of the whole-cell lysate and resuspension with 30 ml of wash buffer I (25 mM Tris, pH 8.0, 300 mM NaCl, 1% Triton X-100). The suspension was homogenized until an even consistency was obtained. The inclusion bodies were then washed twice by centrifugation and homogenization in wash buffer I. Three subsequent washes with wash buffer II (25 mM Tris, pH 8.0, 300 mM NaCl) were performed to remove the excess Triton X-100. Purified inclusion bodies were stored at −80ºC until required. Proteins were resolubilized in 25 mM Tris, pH 8.0, 250 mM NaCl, 6 M guanidine hydrochloride, 2 mM beta-mercaptoethanol by rotation at 4ºC before centrifugation. Harvest supernatant was applied to nickel-nitrilotriacetic acid resin and purified via metal affinity chromatography. Purity of the denatured purified protein was assessed via SDS-PAGE and Western blotting.

Denatured TLN4_209–1295_ was diluted to a final concentration of <1 mg/ml and applied dropwise to 100 ml of refold buffer (50 mM Tris, pH 8.2, 250 mM NaCl, 0.5 M arginine, 0.44 M sucrose, 4 mM reduced glutathione, 0.4 mM oxidized glutathione, 0.05 mM Tween 20) that was constantly stirring at 4ºC. Refolding proceeded for 4 h at 4ºC with gentle stirring. The refolded protein was then dialyzed overnight against 25 mM Tris, pH 8.0, 300 mM NaCl at 4ºC. Postdialysis, the protein was concentrated to approximately 0.5 ml before application to a Superdex 200 10/300 column for further purification via size-exclusion chromatography.

### TLN4 proteolytic activity.

Hydrolysis of insulin B chain was measured via reverse-phase high-performance liquid chromatography (HPLC) following incubation of TLN4 (160 μg/ml) with insulin B chain (42 μM; Sigma-Aldrich) in 10 mM Tris for 88 h. HPLC was carried out in a Vydac C_4_ HPLC column using a linear gradient from 0.1% trifluoroacetic acid (TFA) in 95% water, 5% acetonitrile to 0.1% TFA in 50% acetonitrile-water at a flow rate of 1 ml/min. Hydrolysis products were detected at 214 nm and collected for analysis. The obtained peptide products were analyzed on an Applied Biosystems 4800 matrix-assisted laser desorption ionization tandem time-of-flight proteomics analyzer at the University of Kentucky Proteomics Core.

### Parasite culture, transfection, and selection.

T. gondii tachyzoites were maintained by growth on monolayers of human foreskin fibroblasts (HFF) in Dulbecco’s modified Eagle’s medium (DMEM) containing 10% cosmic calf serum (GIBCO), 2 mM glutamine, 10 mM HEPES, 50 μg/ml penicillin-streptomycin (D10 Complete). To generate the TLN4 KO, a knockout construct (previously described in reference 22) containing 1.5 kb of TLN4 5′ and 3′ flanking regions was used, and the HXGPRT selectable marker was replaced with the DHFR-TS selectable marker cassette. This construct was transfected into RHΔ*ku80*Δ*hxg* parasites with a Bio-Rad Gene Pulsar II with 1.5-kV voltage, 25-μF capacitance, and no resistance setting. Pyrimethamine selection was applied the day after transfection, and clones were isolated by limited dilution in 96-well plates. Knockout clones were tested for proper 5′ integration, 5′-AGTTGCAGCCAGAGGCAGAAGCAAGTCC-3′ (a) and 5′-CAGTCAGATAACAGGTGTAGCG-3′ (b), and for proper 3′ integration, 5′-GCGGGTGACGCAGATGTGCGTGTATCC-3′ (c) and 5′-GAAAAGTGTCTGCGTGTTAGCAGC-3′ (d). Knockout clones that were complemented with the TLN4.HA construct were identified with primers TLN4.322.F (e), 5′-GGCTTTTCTGCTTCGTCAAC-3′, and TLN4.1910.R (f), 5′-AAGAGCAGTGGGCTGAAAAA-3′.

The TLN4-IA^1^-HA^2^ complementation plasmid was previously described in reference 22. This plasmid (50 μg) was cotransfected with 5 μg of the pDHFR-TS plasmid containing the selectable marker dihydrofolate reductase-thymidylate synthase by electroporation and drug selection with pyrimethamine. Positive clones were identified by PCR and Western blot analysis.

### Invasion assays.

Invasion assays were performed as previously described (32). Briefly, 1 × 10^7^ parasites were used to infect subconfluent HFF monolayers in 8-well chamber slides for 20 min before fixation with paraformaldehyde. Slides were differentially stained with anti-SAG1 antibodies to differentiate attached versus invaded parasites.

### Egress assays.

Induced egress was performed as described previously (15). Briefly, parasites grown in HFFs in an 8-well chamber slide for 30 h were treated with 1% dimethyl sulfoxide, 2 μM A23187, or 200 μM zaprinast in assay buffer (Hanks' buffered salt solution containing 1 mM CaCl_2_, 1 mM MgCl_2_, and 10 mM HEPES) and incubated for 2 min in a 37°C water bath. Egress was stopped by addition of 2× fixative (8% formaldehyde in 1× PBS). Immunofluorescence was performed with rabbit anti-SAG1 to identify parasites and mouse anti-GRA7 (a generous gift from Peter Bradley) to identify the parasitophorous vacuole membrane. At least 10 fields of view (400× total magnification) per condition were enumerated as occupied or unoccupied.

LDH egress assays were performed as previously described (20). Briefly, parasites were grown in HFF monolayers in 96-well plates for 30 h. Wells were washed with Ringer’s buffer and then treated with 100 μM zaprinast diluted in Ringer’s buffer. Plates were incubated at 37ºC for 20 min before removal and placement on ice. Fifty microliters of the supernatant were removed and centrifuged in a separate round-bottom plate. Thirty microliters of supernatant was subsequently removed and release of lactate dehydrogenase was determined using an LDH cytotoxicity colorimetric assay kit (BioVision).

### Plaque and replication.

Parasites were inoculated into wells of a 6-well plate and allowed to replicate undisturbed for 7 days. The wells were then stained with 0.2% crystal violet for 5 min and rinsed with double-distilled water (ddH_2_O). Images of the wells were scanned, and plaque number and size were analyzed with Image J.

For the replication assay, cells of an 8-well chamber slide were inoculated with 1.25 × 10^5^ tachyzoites and allowed to invade and grow for 17 h and 26 h prior to fixation and indirect immunofluorescence.

### Live gliding video microscopy.

Glass-well dishes (MatTek) were coated with 50% fetal bovine serum (FBS)–50% PBS overnight at 4ºC or 1 h at 37ºC and washed with PBS before use. Parasites were filter purified and resuspended in HHE (Hanks’ salt solution, 10 mM HEPES, EDTA). Parasites were then added to coated dishes and allowed to settle for 5 min at room temperature. Dishes were then moved to a 37ºC chamber with 5% CO_2_ and warmed for 5 min prior to starting video recording. Each video consisted of 1 frame/s recorded for 90 s. Enumeration of the types of gliding motility were carried out by examining the videos in addition to maximum projection images generated by the Zeiss AxioVision software.

### Mouse infections.

All laboratory animal work in this study was carried out in accordance with policies and guidelines specified by the Office of Laboratory Animal Welfare, the U.S. Department of Agriculture, and the American Association for Accreditation of Laboratory Animal Care (AAALAC). The University of Michigan Committee on the Use and Care of Animals (IACUC) approved the animal protocol used for this study (Animal Welfare Assurance A3114-01, protocol no. PRO00008638). Freshly egressed parasites were filter purified in PBS, washed, counted, and injected intraperitoneally in 200 μl of PBS into 6- to 8-week-old female Swiss Webster mice. The experiment was performed once with 4 groups of 12 mice each infected with 10 or 100 tachyzoites of WT or Δ*tln4* parasites. Mice were given water and food *ad libitum*, monitored twice daily, and were humanely euthanized upon showing signs of morbidity.

### Western blotting.

Parasite lysates were generated by filter purifying parasites followed by centrifugation, 1× wash with cold PBS, and resuspension in >90ºC 1× sample buffer. Lysates were boiled for 5 min prior to running on SDS-PAGE gels. Gels were semidry electroblotted (Bio-Rad) onto polyvinylidene fluoride membranes and sequentially probed with mouse anti-TLN4 (22) and horseradish peroxidase-conjugated goat anti-mouse (Jackson ImmunoResearch). Bands were revealed by enhanced chemiluminescence with SuperSignal West Pico Plus chemiluminescent substrate (Thermo Fisher Scientific) and documented with a Syngene Pxi imaging system.

### PLP1 processing.

Extracellular parasites (Δ*ku80*Δ*hxg* [WT], Δ*plp1*, Δ*tln4*, and Δ*tln4TLN4*) were resuspended in PBS in either pH 5.4 or 7.4, and excreted-secreted antigen (ESA) was collected. Pellet lysates and ESA were separated on SDS-PAGE gels, and membranes were probed with antibodies against rabbit anti-PLP1.
